# Capsular Interposition Arthroplasty With Percutaneous Suture Anchoring: A Pragmatic and Novel Surgical Technique for Hallux Rigidus

**DOI:** 10.7759/cureus.8556

**Published:** 2020-06-11

**Authors:** Mark Gilheany, Steven R Edwards, Matthew Cotchett

**Affiliations:** 1 Surgery, Australasian College of Podiatric Surgeons, Melbourne, AUS; 2 Podiatry, La Trobe University, Bundoora, AUS; 3 Podiatry, La Trobe University, Melbourne, AUS

**Keywords:** hallux rigidus, interposition arthroplasty, first metatarsophalangeal joint, hallux disorders, arthroplasty

## Abstract

There is support for the use of capsular interposition arthroplasty (CIA) as an alternative to arthrodesis in the surgical treatment of hallux rigidus. In this technical report, the authors describe novel technical variations to the traditional capsular interposition arthroplasty that are anatomically rational, reproducible, and efficient. This technique was developed by the primary author and has been the preferred approach to CIA for over 10 years. The key technical differences of the procedure are metatarsal head preparation, the use of a proximally based capsular graft, and anchoring by utilizing a simple percutaneous anchoring technique. This approach maintains anatomic joint morphology, preserves vascular supply, and bone stock to facilitate future procedures if required.

## Introduction

Hallux rigidus (HR) involves osteoarthrosis or the impingement of the first metatarsophalangeal joint (MTPJ) and can have a significant impact on an individual’s quality of life. HR affects around 44% of people over the age of 80 years and is the most common condition affecting the first MTPJ after hallux valgus (HAV) [1-2]. HR presents with pain and reduced joint range of motion, especially on dorsiflexion. The severity of HR is established through clinical and radiographic evaluation [2]. HR exhibits a higher level of pain and dysfunction when compared to HAV [3].

Although the symptoms of HR may be managed conservatively via rigid shoes, orthoses, non-steroidal anti-inflammatory drugs (NSAIDs), intra-articular injections, and activity modification, non-operative treatments may fail to improve symptoms and surgical intervention can be considered [4]. Philosophical approaches towards surgery for HR vary significantly [5].

Modern clinical practice guidelines classify HR surgery into two categories: joint salvage and joint destructive procedures [6]. Joint salvage procedures include cheilectomy and chondroplasty, various metatarsal and phalangeal osteotomies, and arthroplasty procedures, including soft tissue interposition [7-11]. Joint salvage procedures aim to reduce pain, increase dorsiflexion, maintain plantarflexion power, and maintain the stability of the first MTPJ whilst concomitantly maintaining the length of the first metatarsal and hallux to prevent transfer metatarsalgia [12]. Not surprisingly, achieving these parameters in one surgical episode can be difficult [6]. Joint destructive procedures include resection arthroplasties, partial and total joint replacements, and arthrodesis [13-14]. Arthrodesis is unique, as the primary aim is pain relief without preservation of motion. It has been described as a preferred technique; however, the loss of motion is cited as a concern [13,15-16].

Capsular interposition arthroplasty (CIA) involves the removal of the osteophytic bone (cheilectomy) and the interposing of a split-thickness autogenous (MTPJ joint capsule) graft to re-surface the metatarsal head. Other graft tissue types have been described such as hamstring and synthetic variants [17]. The use of an interposition graft has been studied extensively, including in three systematic reviews [6,17-19]. These reviews highlight both the potential value of the procedure and concerns regarding the lack of a standardized approach to the procedures [2,6,12].

This lack of a standardized approach is due in part to the inherent technical difficulties associated with this procedure. Such difficulties include concerns with the stabilization of the graft, achieving adequate metatarsal head coverage, and blocking of the joint through a lack of decompression [17]. These difficulties and the variations in technique may be enough for some surgeons to refrain from using this procedure. This study aims to address these difficulties by describing a pragmatic and reproducible means to perform the procedure.

## Technical report

Incision

A standard dorsomedial approach to the first MTPJ can be used. Sharp and blunt dissection are performed to reflect the subcutaneous fascia from the deep fascia whilst concomitantly protecting the integrity of the first MTPJ and extensor hood tissues.

Key Technical Difference

The primary author (MG) prefers a short dorsomedial curvilinear incision that facilitates access to the lateral base of the proximal phalanx and minimizes scar adhesion at the dorsal joint line.

Capsular dissection and joint exposure

Following the identification of the extensor tendon and the joint borders, a full thickness deep fascia incision is performed along the medial lower-third of the first MTPJ superior to the metatarso-sesamoid articulation, reflecting the periosteum and capsular tissues.

Key Technical Difference

The medialized deep fascia incision of this technique allows for an increased autogenous flap diameter that interposes over the entirety of the metatarsal head, reducing the risk of devascularization of the flap.

Joint inspection

**Figure 1:**
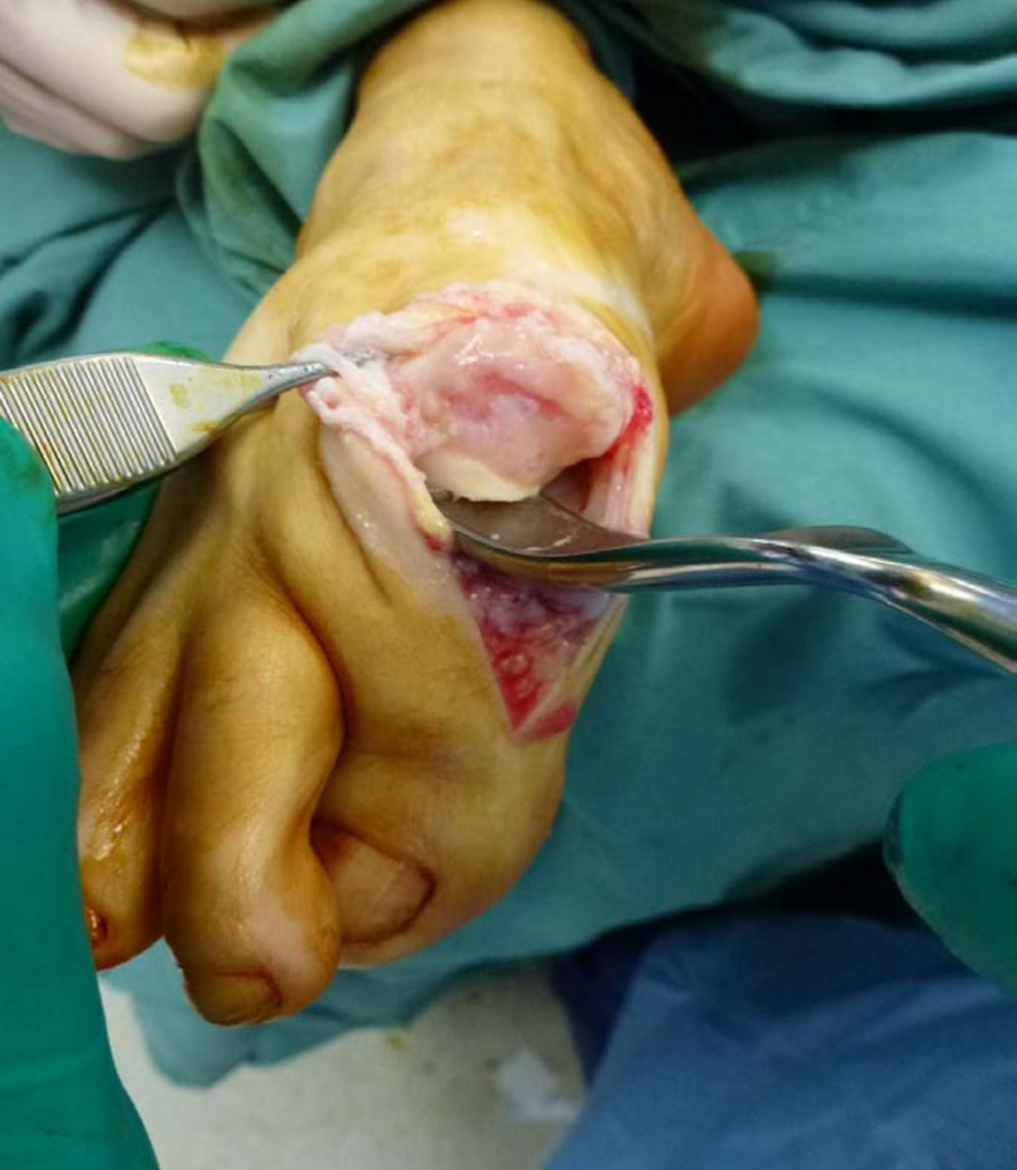
Severe degenerative joint disease with a full thickness osteochondral defect of the first metatarsal head

The MTPJ can be directly visualized at this point (Figure 1) and a decision made whether to perform a CIA or an alternative procedure.

Anatomic osseous re-modeling - metatarsal head

The medial, dorsal, and lateral exostoses surrounding the metatarsal head are resected (cheilectomy). This is similar to a modified Stone resection [12]. The metatarsal head chondral plate is removed using rongeurs as if preparing for arthrodesis. Any dystrophic flaps of cartilage and plica are removed, followed by the anterior-dorsal contouring of the metatarsal head until parabolic morphology is achieved. Healthy crista and plantar cartilage is not removed as long as an adequate range of motion of the joint is achievable. This amount of metatarsal head resection should allow for normal weight-bearing between the sesamoids and metatarsal head and preservation of the intra-osseous blood supply.

Key Technical Difference

**Figure 2:**
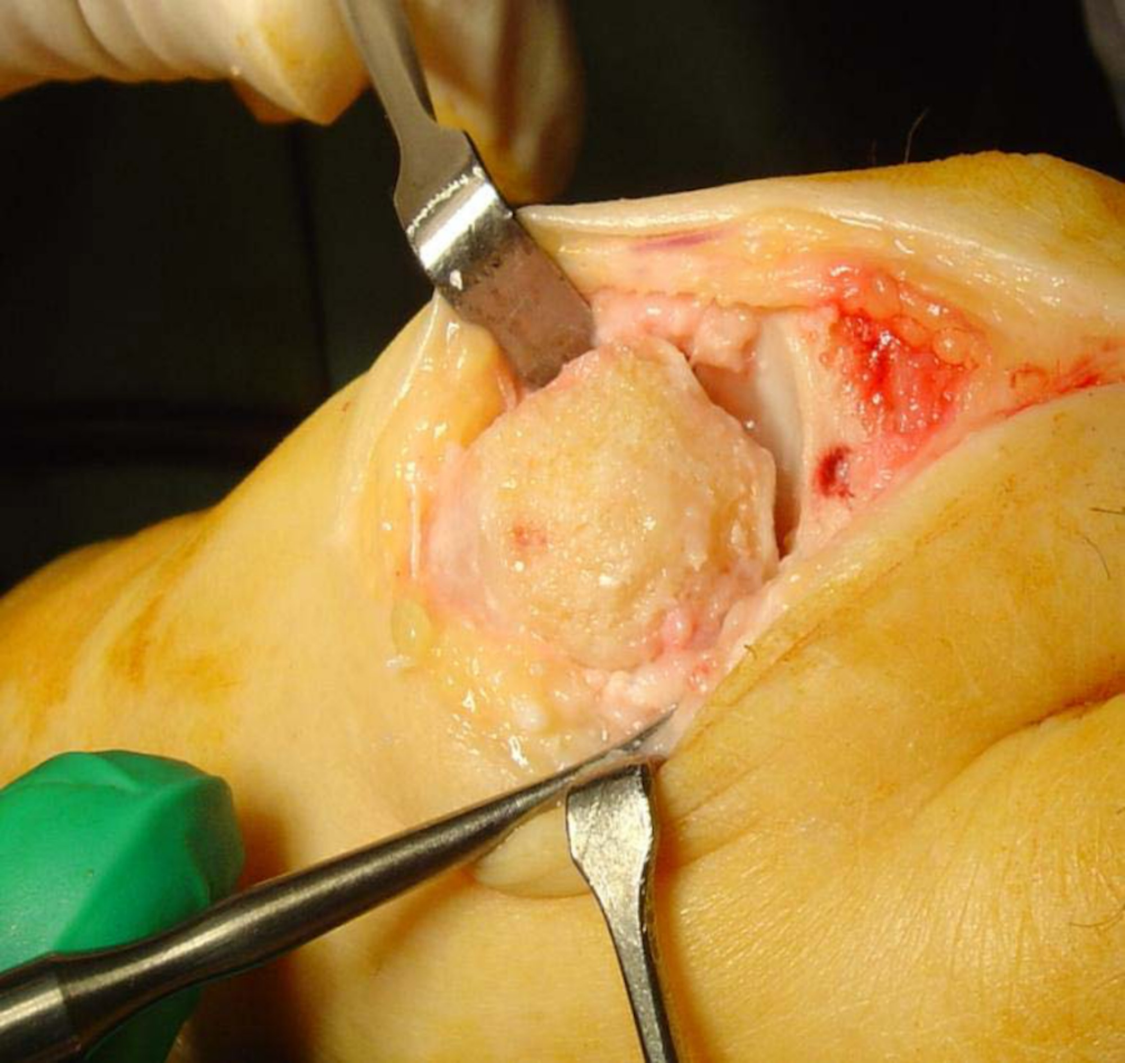
The chondral plate removed in line with the anatomic shape of the metatarsal head

This re-creation of the metatarsal head's anatomic shape provides joint decompression without the need for osteotomy, also eliminating the potential for avascular necrosis (AVN) as a complication. The bleeding subchondral bone aids in the incorporation of the capsular graft and is likely to assist in the maintenance of graft vascularity (Figure 2).

Anatomic osseous re-modeling - sesamoids

The sesamoids are inspected and released from any arthrofibrosis along the plantar aspect of the first metatarsal head. An important step of this technique is the removal of the distal sesamoid and phalangeal base calcific enthesophytes, which, if present, may result in a decreased range of motion postoperatively. Progression to arthrodesis can be initiated if severe sesamoidal cartilage loss is exhibited. If not, CIA can proceed.

Anatomic osseous re-modeling - proximal phalanx

Dorsal, lateral, and medial spurring is resected. In cases of a severe chondral deficit, a ‘silver-dollar Keller’ procedure has been described, which involves the resection of the articular surface and the proximal aspect of the phalanx at a 30-degree angle from the joint line [20]. Ideally, less bone resection than described in this technique is undertaken.

The first MTPJ is then loaded and the range and quality of motion is assessed. Eburnation (compaction at uneven wear points) can be seen where a blockage is occurring. Additional contouring and decompression of both surfaces is performed until normal alignment and a functional parabola with a smooth range of motion is achieved without uneven wear points. The wound is then irrigated of any surgical debris.

Capsular interposition - preparation and placing of the proximal flap

**Figure 3:**
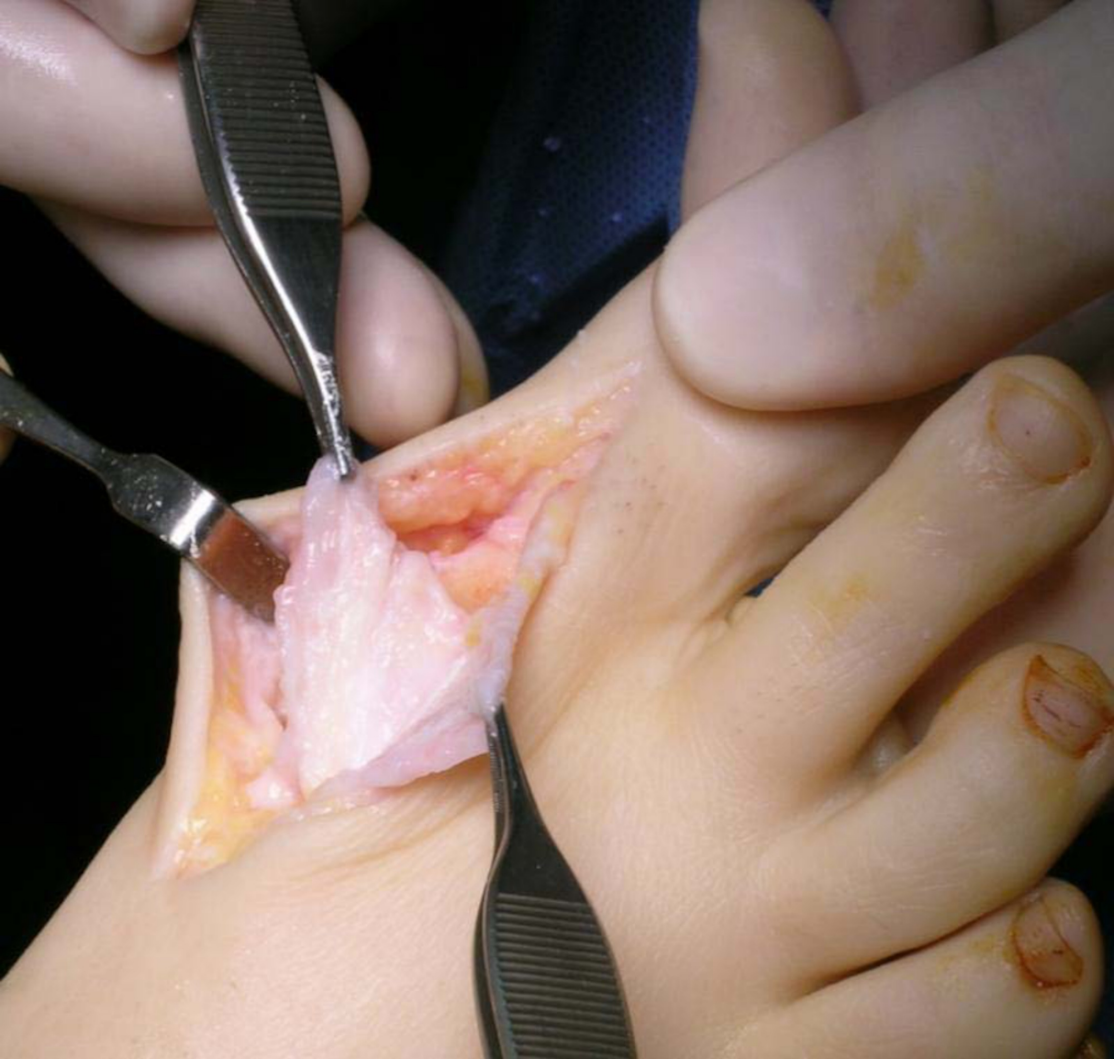
Harvesting of the full thickness capsular flap

The deep fascia incision is extended distally over the base of the proximal phalanx. Extensor hallucis longus (EHL) is protected whilst the capsular insertion and extensor hallucis brevis (EHB) are harvested. The graft is obtained by separating the dorsal capsular tissues from the distal aspect of the EHB tendon and the distal aspect of the extensor hallucis capsularis (EHC) tendon from the overlying EHL tendon, tendon sheath, and extensor hood tissues. This creates a semi-circular tenocapsular flap. The flap is then dissected dorsally from the underlying metatarsal head and the joint is further inspected (Figure 3). These tissues consistently demonstrate marked hypertrophy in HR, allowing for a thick, spongy, and resilient autogenous graft that can resist the pressure, stretching and shearing forces of the first MTPJ [18].

Key Point

The proximally based flap is always contiguous with proximal capsular and periosteal tissue. It is harvested with care to maintain its dorsolateral insertions to protect vascular in-flow.

**Figure 4:**
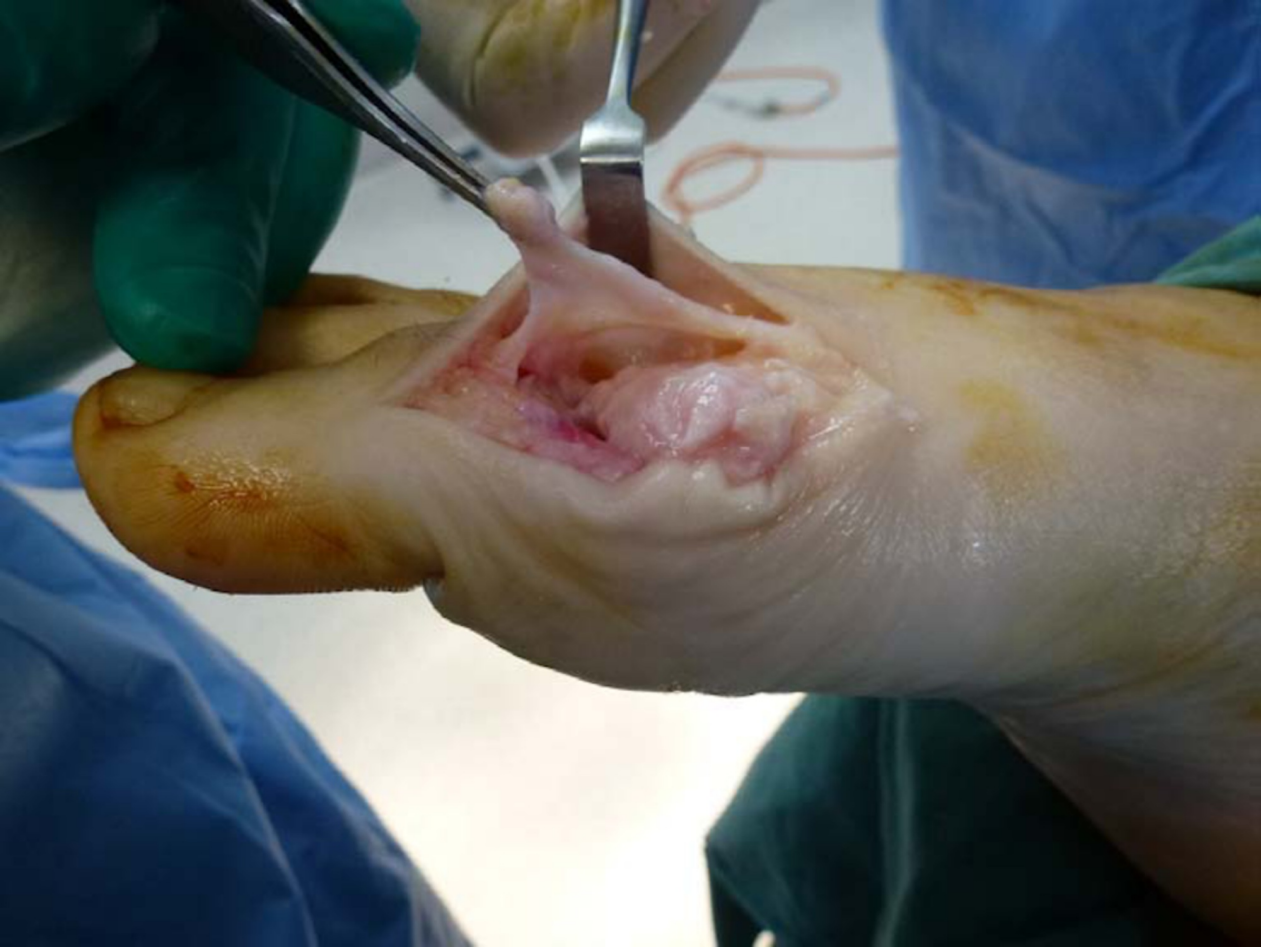
The extensor hood is separated from the underlying joint capsule and the deep capsule layer is tagged to maintain anatomic position.

Attention is then directed to the preparation of a viable flap to interpose over the metatarsal head. Initially, the EHL tendon contained within the hood ligament is diligently separated from the underlying capsule and tendon tissue. This dissection technique may require an assistant to hold the full thickness deep fascial flap under tension to assist in the identification and separation of tissue planes whilst maintaining the integrity of both layers (Figure 4). Once the dorsal capsular flap is freed from the extensor tendon complex, its distal ends are inserted into the joint space. The flap is then advanced anteriorly until it covers the head of the first metatarsal in toto. The correct length of the flap should allow seating at the plantar distal aspect of the metatarsal head. If the capsule length is not adequate, further decompression can be carried out dorsally about the metatarsal head or the capsular proximal attachments freed.

**Figure 5:**
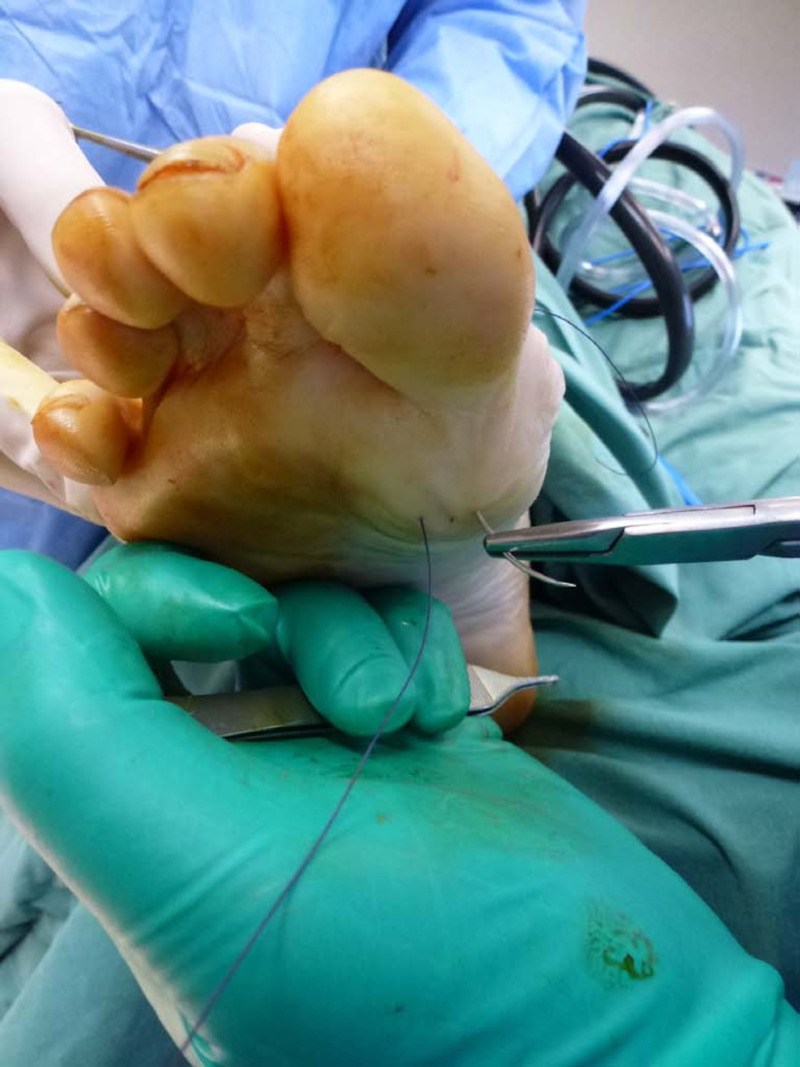
The distal end of the capsular flap pulled over the metatarsal head and tied percutaneously laterally and medially

Once adequate flap length is attained, the distal medial and lateral ends are secured with 2-0 absorbable sutures using Kessler-Kleintert knots [12]. The needle is then bent to decrease its curvature to a one-fifth circle and then advanced plantarly into the joint space and brought through the plantar skin distal to the tibial sesamoid. The needle on the lateral side is advanced plantarly through the skin distal to the fibular sesamoid (Figure 5).

Key Technical Difference

This differs from the standard CIA technique where the capsule is sutured to the plantar plate or another internal structure [6].

**Figure 6:**
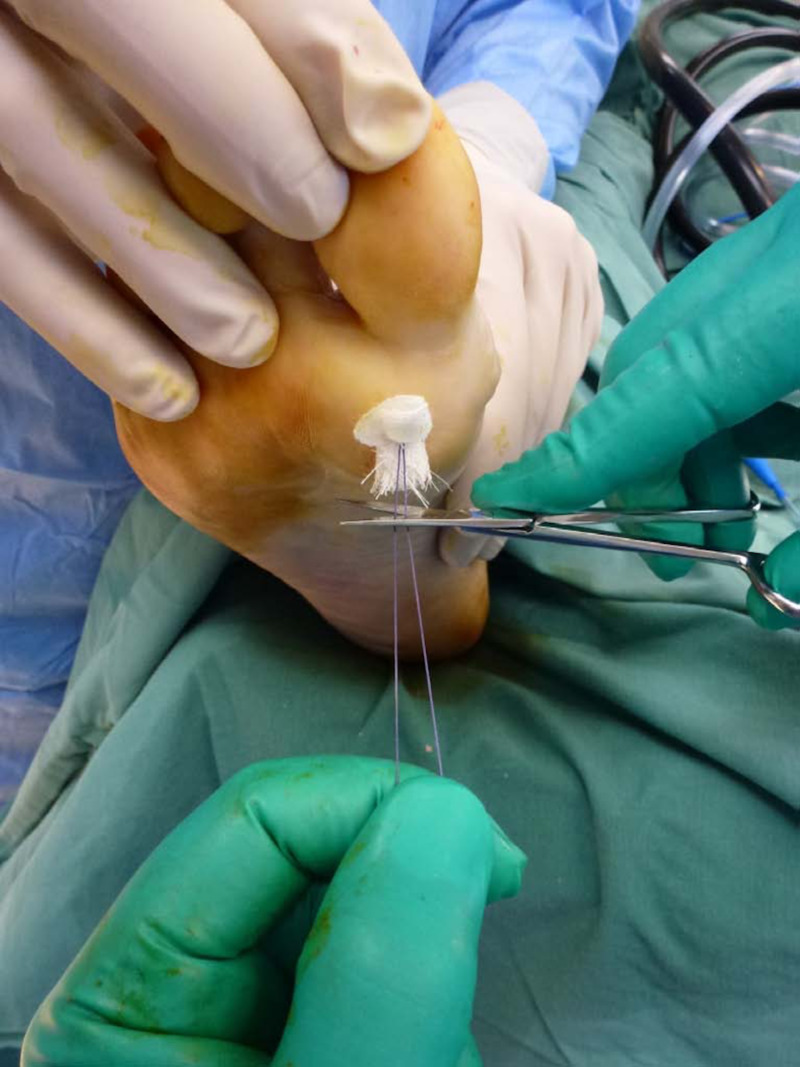
Tension is applied to the suture whilst the knot is tied off and a square piece of gauze is used to protect the skin.

**Figure 7:**
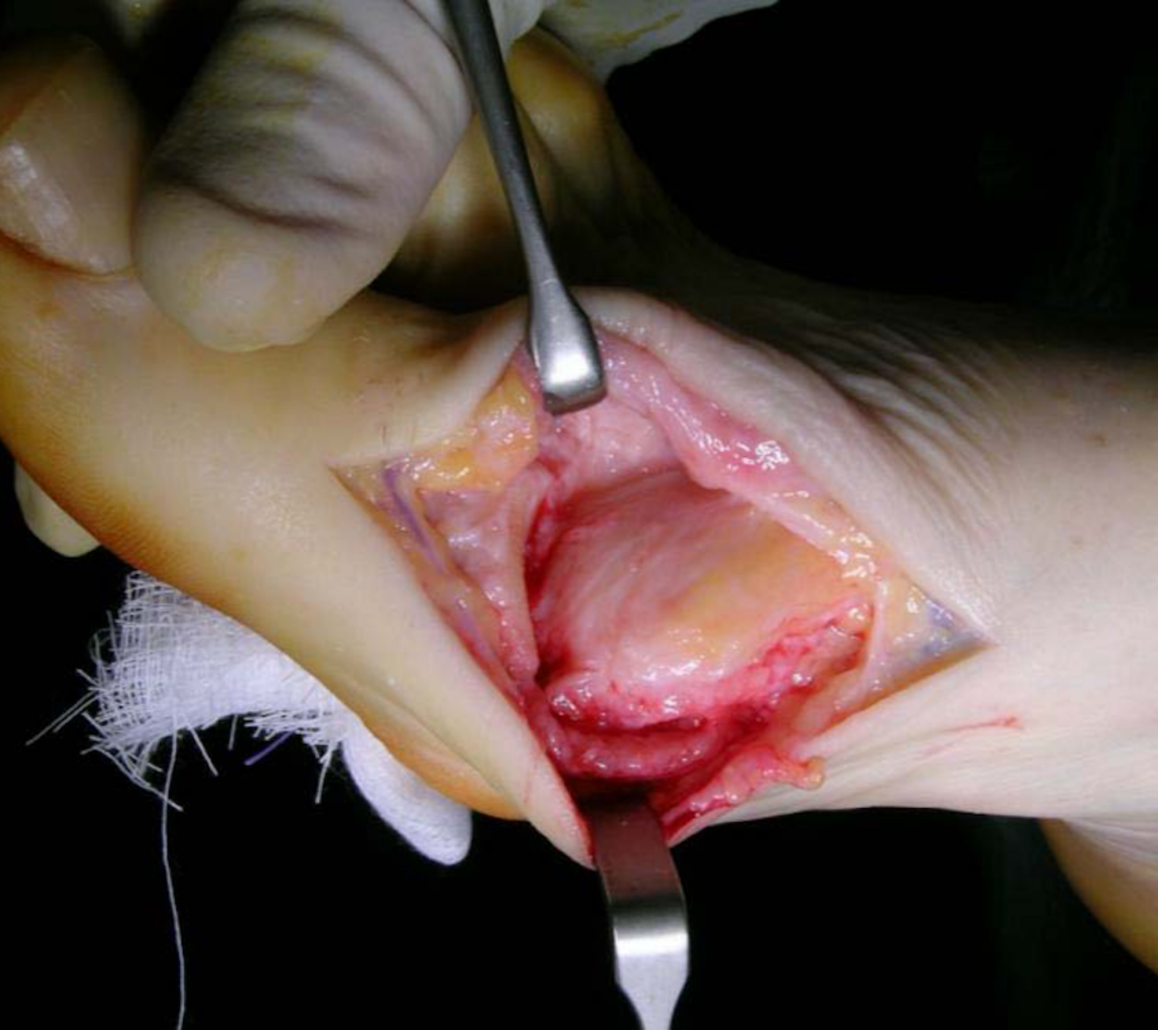
Full coverage of the metatarsal head is exhibited dorsally and within the joint space.

To aid the advancement of the capsular flap, an assistant may use a periosteal elevator to distract the flap distally and plantarly whilst the surgeon distracts the two sutures through the plantar surface of the foot with tension during knot tying (Figure 6). These percutaneous sutures should be parallel to each other, at an equal distance away from the metatarsal head, and tied to each other over a square piece of gauze to protect the plantar skin. The metatarsal head is then inspected to ensure full coverage of the capsular flap (Figure 7).

Final closure

**Figure 8:**
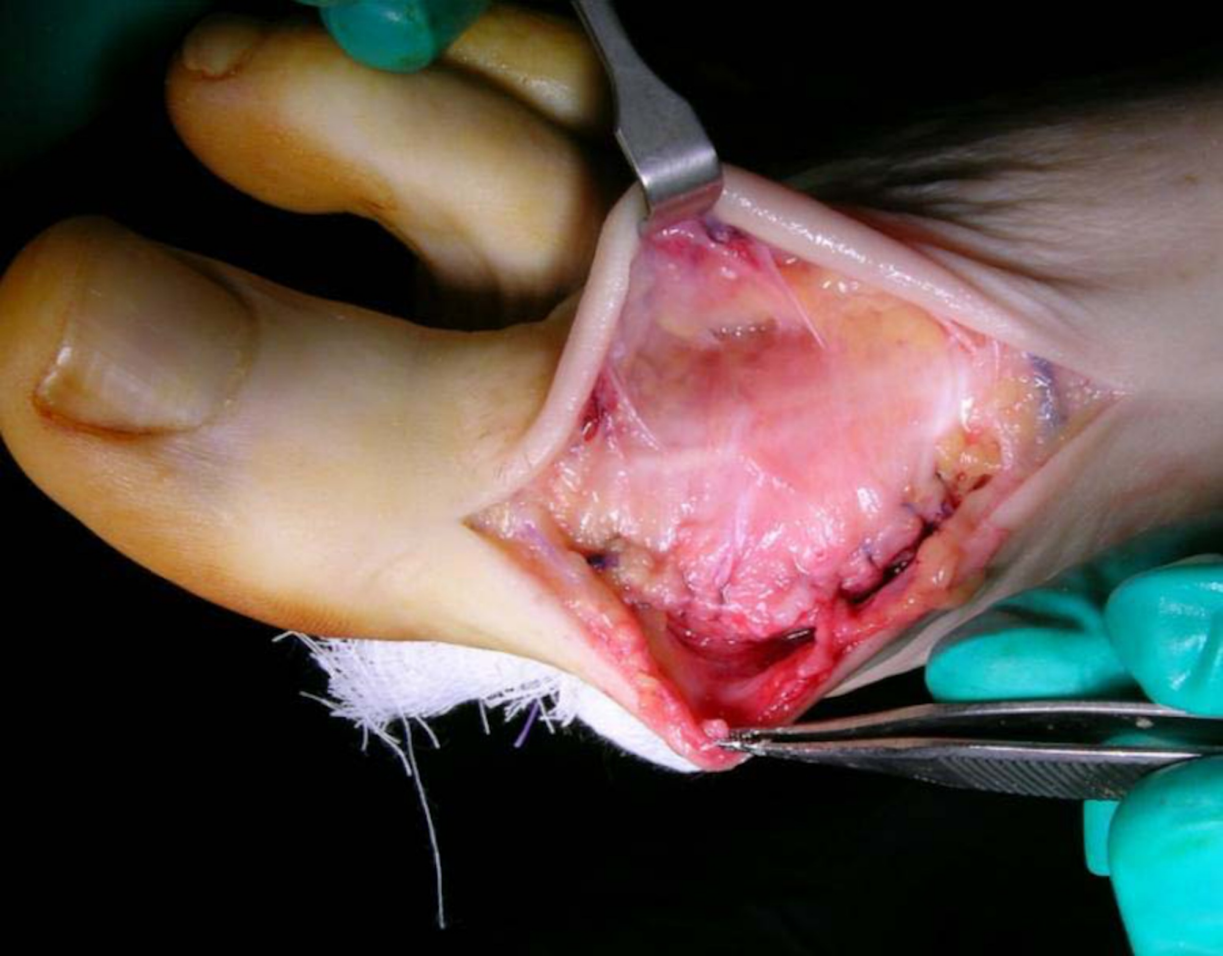
The extensor hood fibers covering the EHL tendon are identified and re-approximated EHL: extensor hallucis longus

The deep capsular flap is secured medially as a standard capsular closure at the level of the metatarsal head to maintain anatomic position utilizing a 3-0 absorbable suture. The extensor hood fibers are then approximated (Figure 8) with a running horizontal mattress 2-0 absorbable suture. Deep closure should be anatomic. Skin closure is performed as per the surgeon's preference.

Postoperative phase

A modified Jones compression bandage is applied and the patient is allowed immediate, protected weight-bearing within a rigid-soled postoperative shoe. The percutaneous sutures are removed and gentle range of motion exercises are initiated at the third postoperative week and the patient begins to return to footwear once these sutures are removed.

**Figure 9:**
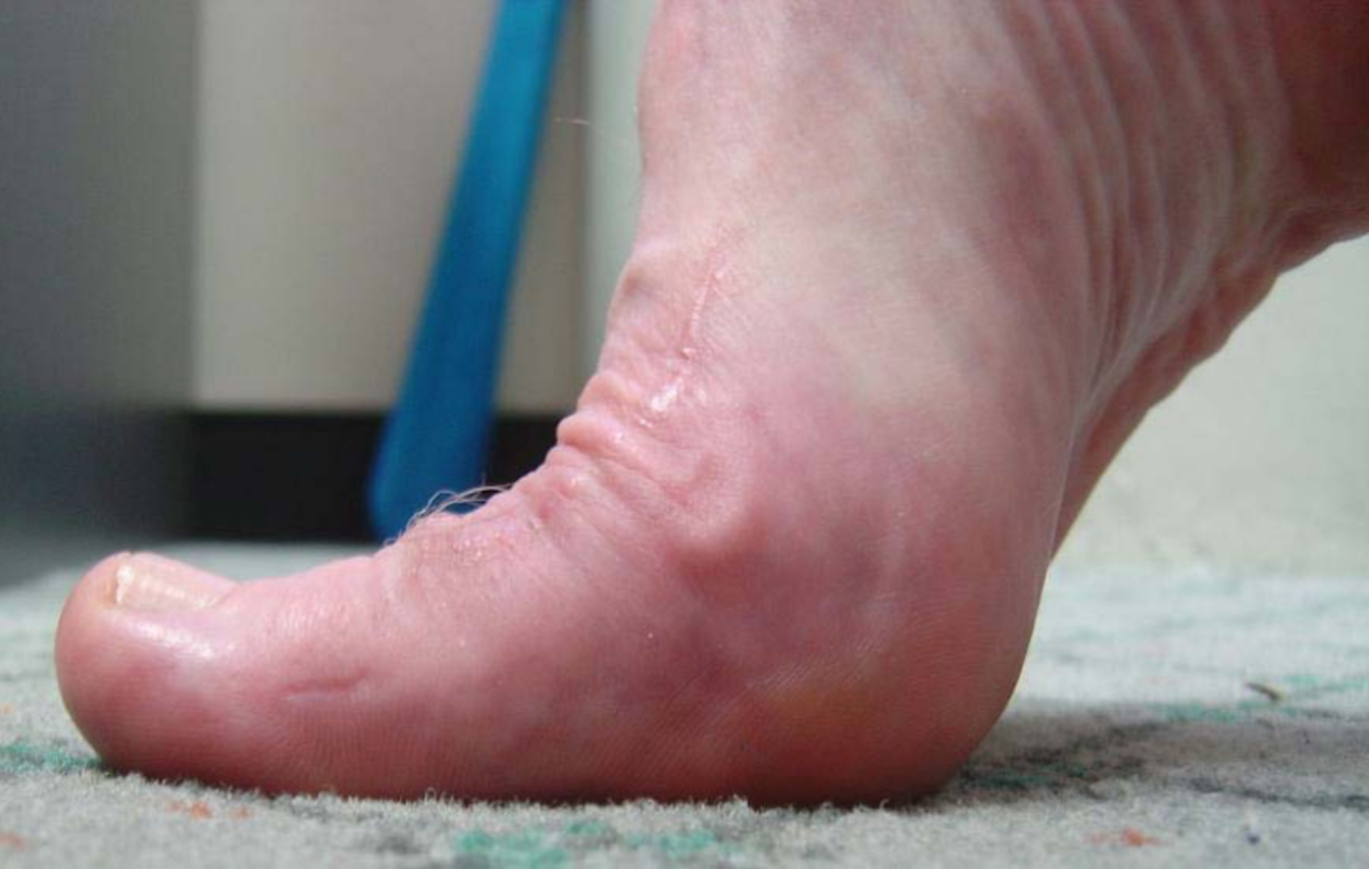
Clinical review six months postoperatively demonstrating pain-free first metatarsophalangeal joint range of motion

**Figure 10:**
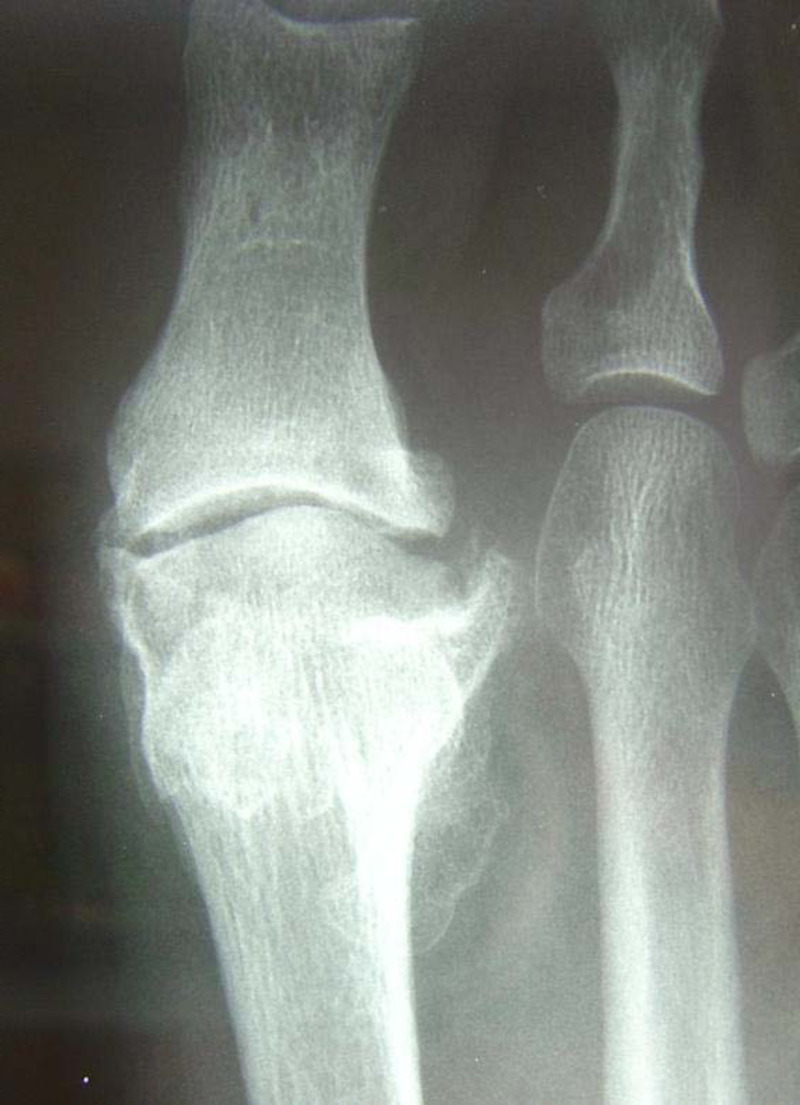
Preoperative plain film radiographs showing degenerative change

**Figure 11:**
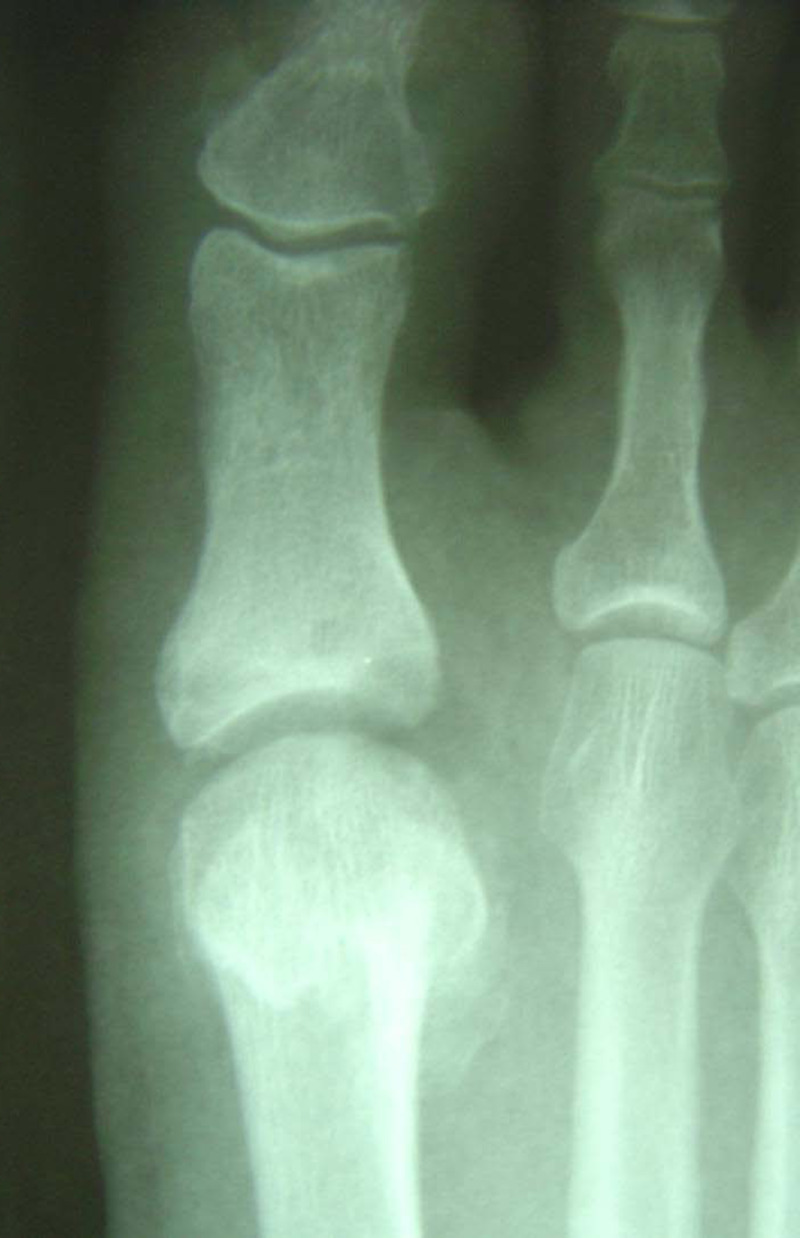
Six weeks postoperative radiographs demonstrating joint decompression

In our experience, patients demonstrate some edema and limitus around the sixth week, which may persist for up to several months. To address this, range of motion exercises and gait re-training are instituted, with acceptable dorsiflexion and hallux purchase generally observed six months postoperatively (Figure 9). Preoperative (Figure 10) and postoperative (Figure 11) radiographs demonstrate joint decompression with anatomic alignment.

Precautions

This procedural variation is not without potential pitfalls. Slight plantarflexion of the hallux is recommended whilst the joint capsule is re-approximated in order to ensure adequate hallux ground purchase is achieved postoperatively. The plantar suture exit points require protection over the first three weeks postoperatively with off-loading padding. It is common to observe a small compression indentation once the percutaneous plantar sutures are removed, however, this resolves within a short time period. The authors note a single case of secondary infection and wound breakdown occurring in this location, which resolved with oral antibiotic therapy.

From four weeks postoperatively, appropriate rehabilitation exercises to ensure the maintenance of the first MTPJ range of motion specific to this procedure appear to improve the rate of recovery.

Transfer metatarsalgia has been identified as the primary complication of first MTPJ surgery, occurring in 13.6% to 57% of patients according to a recent systematic review, however, these figures may contain confounding errors, as studies included outdated surgical techniques [19].

Interestingly, instances of avascular necrosis (AVN) of the metatarsal head with CIA appear significantly lower (5.4%) as compared to other common distal first metatarsal procedures such as the chevron osteotomy 11%-50% [20]. This technique does not breach the arterial supply to the first metatarsal head [20].

**Figure 12:**
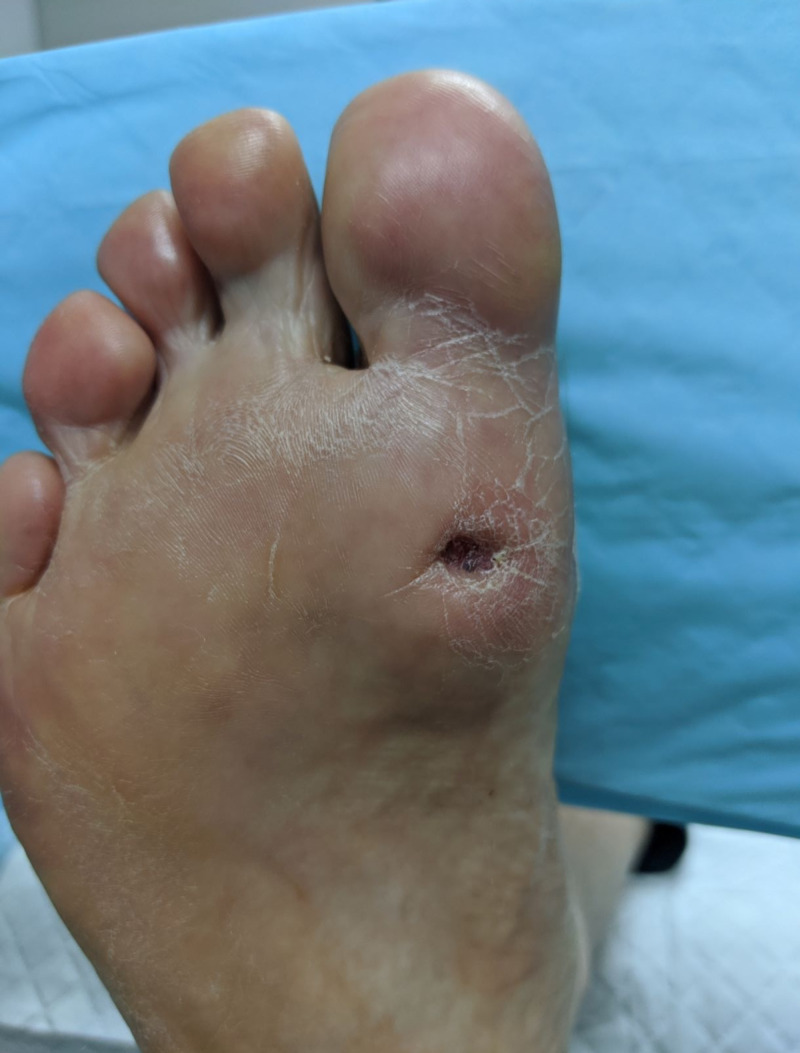
Indentation caused by the percutaneous suture anchor. This appears to resolve within a few days of suture removal and off-loading.

Compression indentation (Figure 12) caused by the percutaneous suture anchor has been observed in occasional cases by the lead author, however, these resolve within a few days of suture removal and off-loading.

## Discussion

There is support in the literature for CIA as a viable alternative to fusion, with studies consistently showing an improved range of motion and function with pain reduction [6,10,12,15]. However, the lack of a consistent approach to this procedure limits the generalizability of it as a potential approach for HR.

**Figure 13:**
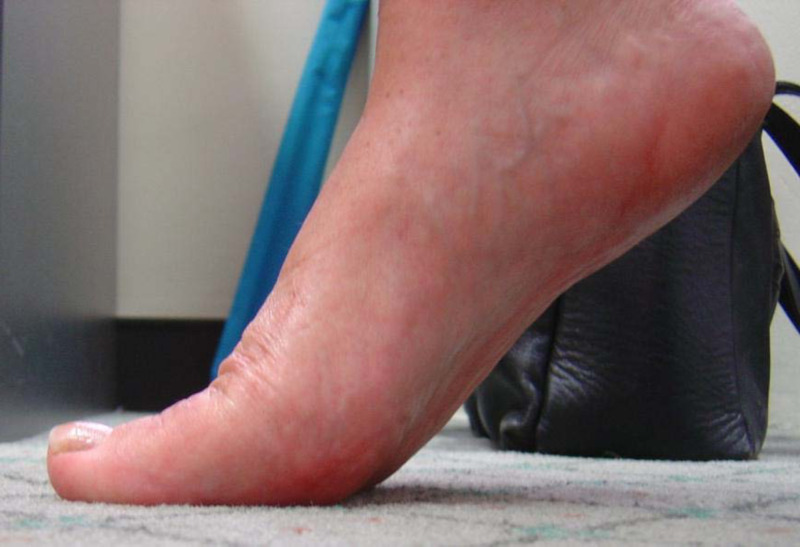
Clinical view five years postoperatively

Barriers to a standardized approach include the difficulty of reliably anchoring the graft over the metatarsal head, as the suturing of the graft to the plantar plate whilst achieving total metatarsal head coverage can be challenging. We present a variation to address this difficulty. Our variation of the CIA involves percutaneous suturing of the graft through the plantar skin of the first MTPJ as a stabilizing mechanism to facilitate attachment to a vascular bed of bleeding subchondral bone. We believe this provides a safe and reliable advancement in the performance of this procedure, provides a viable surgical solution for stage 2 and 3 HR, with some evidence that it may have a place in stage 4 of the deformity [17]. This approach is technically straightforward and preserves the anatomic (osseous and vascular) integrity of the first MTPJ. It overcomes concerns regarding soft tissue flap quality and the inherent difficulties with its anchoring. Significantly, the requirement for osteotomy to decompress the joint is also removed and a vascular bed is created for the graft. Maintenance of the normal anatomic contour of the bone surfaces reduces complications associated with revision surgery, such as arthrodesis, whilst retaining the first MTPJ joint movement (Figure 13). When this procedure has been performed, arthrodesis can still be performed if required, without the need for bone grafting.

## Conclusions

This variation of the CIA procedure is anatomically rational, reproducible, and efficient and has been the preferred approach of the primary author for over 10 years. It differs from a traditional CIA via metatarsal head preparation, a proximally based capsular graft, and novel anchoring utilizing a simple percutaneous anchoring technique. This approach maintains anatomic joint morphology and preserves vascular supply and bone stock to facilitate future procedures if required.
